# Cannabis use is associated with alterations in NLRP3 inflammasome related gene expression in monocyte-derived macrophages from people living with HIV

**DOI:** 10.3389/fimmu.2025.1634203

**Published:** 2025-11-07

**Authors:** Kyle C. Walter, Bryant Avalos, Mary K. Ford, Anna E. Laird, Ali Boustani, Matthew Spencer, Leeann Shu, Antoine Chaillon, Melanie Crescini, Debralee Cookson, Ronald J. Ellis, Scott L. Letendre, Jennifer Iudicello, Jerel Adam Fields

**Affiliations:** 1 Department of Psychiatry, University of California, San Diego, La Jolla, CA, United States; 2 Department of Medicine, University of California San Diego, La Jolla, CA, United States; 3 Department of Neurosciences, University of California San Diego, La Jolla, CA, United States

**Keywords:** NLRP3 inflammasome, HIV-associated neuroinflammation, cannabis, CBD, monocyte-derived macrophages

## Abstract

**Introduction:**

Human immunodeficiency virus (HIV) infection is often associated with chronic inflammation and cognitive dysfunction in people living with HIV (PWH). The nucleotide-binding oligomerization domain-like receptor containing pyrin domain 3 (NLRP3) inflammasome plays a crucial role in the secretion of pro-inflammatory cytokines, specifically interleukin (IL)-18 and IL-1β. Cannabis use and certain phytocannabinoids, such as cannabidiol (CBD), may provide therapeutic benefits in conditions associated with chronic inflammation.

**Methods:**

In this cross-sectional study, we investigated the relationship between cannabis use and *NLRP3*-related gene expression in monocyte-derived macrophages (MDMs) from PWH (n = 43) and people without HIV (PWoH; n = 22). Participants were categorized as naïve, moderate, or daily cannabis users. Donor-derived MDMs were treated with CBD (30 μM), IL-1β (20 ng/mL), or CBD + IL-1β for 24 hours to examine effects on *NLRP3*-related gene expression. Gene expression data were analyzed using one-way and two-way ANOVA with Holm-Sidak’s multiple comparisons tests. Correlations between gene expression and clinical parameters were assessed using Pearson's correlation coefficient. Statistical significance was determined at p < 0.05.

**Results:**

MDMs without treatment from PWH exhibited 83% higher *NLRP3* mRNA expression compared to MDMs from PWoH. Furthermore, MDMs without treatment from moderate cannabis users expressed 61% less *IL1β* mRNA compared to naïve users, and MDMs from daily users expressed a 64% increase in *IL18* expression compared to moderate users. Additionally, MDMs treated with CBD and IL-1β showed a 22% decrease in *NLRP3* mRNA expression compared to IL-1β treated MDMs. When treated with CBD and IL-1β, we observed a significant increase in both *IL1β* (3-fold, p < 0.01) and *IL18* (2-fold, p < 0.01) expression compared to vehicle. The relationship between *NLRP3* mRNA expression in MDMs and global deficit scores in PWH not using cannabis was inverse to that relationship in PWH using cannabis.

**Discussion:**

Overall, these findings suggest that CBD, as consumed through cannabis use, may mitigate NLRP3 activation in PWH, potentially offering therapeutic benefits for chronic inflammation. However, the unexpected effects on downstream cytokine mRNA expression, combined with product heterogeneity, underscore the need for future mechanistic studies to fully delineate cannabinoid–inflammasome interactions in the context of HIV.

## 1 Introduction

HIV-1 infection is associated with immune dysregulation, contributing to both disease progression and the development of comorbidities (1). While antiretroviral therapy (ART) has successfully transformed HIV from a fatal disease into a manageable chronic condition (2), neurological complications such as neurocognitive impairment (NCI) remain a major concern (3). While the cause of NCI in PWH is often multifactorial, chronic inflammation within the central nervous system (CNS) is a primary etiology (1, 4–6). Consequently, there is a need to better understand mechanisms of chronic inflammation in people living with HIV (PWH).

Chronic inflammation is characterized by a prolonged, multifaceted, and often maladaptive immune response that underlies the pathogenesis of numerous diseases, including autoimmune disorders (7, 8), cancers (9), and neurodegenerative diseases (10, 11). This persistent inflammatory state is likely driven by a complex interplay of factors, including ongoing viral replication, immune dysregulation, chronic activation of inflammatory pathways, and possibly chronic exposure to ART (12–17). Among these pathways, the nucleotide-binding oligomerization domain-like receptor containing pyrin domain 3 (NLRP3) inflammasome has emerged as a critical mediator of inflammation in PWH. The NLRP3 inflammasome is a critical component of humans’ innate immune response and a potential target for modulating chronic inflammation in PWH (18). NLRP3 is a cytosolic multiprotein complex that detects and responds to a wide array of pathogenic and endogenous danger signals, like adenosine triphosphate and lipopolysaccharides (19). Three proteins form the NLRP3 complex: the NLRP3 sensor protein, an apoptosis-associated speck-like protein containing a caspase recruitment domain adaptor protein, and the effector protein pro-caspase-1 (20–22). Expression of the NLRP3 complex is ubiquitous in most tissues and cell types, even those of non-myeloid lineage (23–30). A wide variety of stimuli such as viral RNA, bacteria, and protozoan pathogens can trigger the active inflammasome NLRP3 complex (31–33). Upon activation, NLRP3 recruits and activates caspase-1, which processes pro-inflammatory cytokines like interleukin (IL)-1β and IL-18 into their active forms, thereby amplifying the inflammatory response (34). These inflammatory cytokines are then released from the cell through membrane pores opened by gasdermin D, another protein that is cleaved and activated by caspase-1 (35). PWH who are immunological non-responders exhibit increased *NLRP3* and *caspase-1* gene expression (36). While essential for host defense against infections, dysregulation of the NLRP3 inflammasome has been implicated in the pathogenesis of various inflammatory diseases, including type 2 diabetes, Crohn’s Disease, Alzheimer’s disease, and atherosclerosis (37–40). HIV-induced cellular stress signals, and ART, make the NLRP3 inflammasome a central player in sustaining chronic inflammation in PWH via the release of inflammasome genes *IL1β* and *IL18*.

There are three distinct types of macrophages in the brain which are critically important in HIV infection, pathogenesis, and immune response (41, 42). Perivascular macrophages and microglia are resident in the parenchyma of the brain (43, 44); While monocyte-derived macrophages (MDMs) are trafficked into the brain after infection occurs using a “Trojan horse” mechanism (45). MDMs often act as latent HIV reservoirs producing HIV proteins and avoiding the cytopathic effects of the virus (46–48). These MDM cells also drive systemic inflammation by secreting pro-inflammatory cytokines such as IL-1β, IL-6, and TNF-α, which disrupt endothelial tight junctions and increase blood brain barrier (BBB) permeability (49, 50). They also release chemokines and neurotoxic factors which trigger neuroinflammation, oxidative stress, and neurotoxic damage (51–53). Additionally, antiretroviral drugs have variable BBB penetration efficiency, which makes these MDMs difficult to target (54–57). Given their resistance to ART and their sustenance of chronic inflammation, MDMs are crucial targets for understanding viral persistence, CNS implications, and the chronic inflammation seen in PWH.

Cannabis use among PWH is common and has shown therapeutic potential for managing HIV-related comorbidities. The cannabinoids delta-9-tetrahydrocannabinol (THC) and cannabidiol (CBD) are known for their anti-inflammatory properties, which may help mitigate neuroinflammation associated with neurodegenerative diseases (58, 59). Recent cannabis use has been associated with lower inflammatory biomarkers in PWH (60); however, the influence of differing cannabis use frequencies on NLRP3 activation and downstream inflammatory pathways in PWH remain poorly understood. These individuals also exhibit reduced circulation of inflammatory biomarkers, decreased viral DNA in tissues, and a lower prevalence of NCI (61–63). Notably, cannabis use among PWH occurs at rates 25% higher than in the general population, emphasizing the importance of understanding its effects on the NLRP3 inflammasome within the context of HIV infection (64).

While cannabis is associated with anti-inflammatory and neuroprotective benefits, the specific pathways through which it exerts these effects in the context of HIV are not yet fully understood. The interplay between the NLRP3 inflammasome and HIV remains an active area of research, with significant implications for understanding how cannabis may modulate immune responses and chronic inflammation in this population. Thus, this study is the first of its kind to investigate the role of the NLRP3 inflammasome in MDMs generated from a cohort of PWH with variable cannabis-use patterns. We propose that varying patterns of cannabis use among PWH may differentially modulate NLRP3 inflammasome activation, leading to alterations in chronic inflammation and subsequent neurological outcomes. Specifically, we hypothesize that PWH who use cannabis at moderate to daily frequencies will express lower levels of *NLRP3* mRNA, compared to non-users. The findings presented here reveal that HIV affects *NLRP3* expression, and this is modulated by cannabis use. Further investigation of the *NLRP3* pathway reveals that cannabis use patterns heavily influence NLRP3-related cytokine response in PWH as well. Insights gained from this research could inform the development of tailored therapeutic strategies for managing chronic inflammation in PWH. Such strategies may ultimately improve health outcomes and quality of life for PWH.

## 2 Materials and methods

### 2.1 Study population

This study recruited PWH (n = 43) and people without HIV (PWoH; n = 22) with varying demographic characteristics (e.g., age, sex, race, education; **Table 1**). Participants were grouped based on their HIV status and cannabis use patterns following recruitment and comprehensive evaluations as part of an NIH-funded and UCSD IRB-approved study conducted at the HIV Neurobehavioral Research Program (HNRP) in San Diego, California, USA. All PWH were on stable ART for at least six months and virally suppressed. Before the assessment, current cannabis users were asked to maintain their regular use pattern. Participants were classified into three cannabis use groups based on consistent cannabis use patterns over the six months prior to assessment: naïve (never used or no use in the past 60 days and low use of cannabis in the past five years [i.e., ≤ 6 times per year]), moderate (1 to 6 days per week), or daily (7 days per week). Participants were administered a comprehensive medical, laboratory (including venous blood collection and urine drug screen), and neurobehavioral assessments. Individuals who tested positive for substances (other than cannabis for the regular cannabis use groups) were excluded or rescheduled to minimize the potentially confounding effects of acute substance use. Additional exclusion criteria include uncontrolled medical, psychiatric, or neurological conditions; comorbidity of infection; a DSM diagnosis of moderate to severe drug use disorder other than cannabis within the past five years, or mild use disorder within the past six months (excluding tobacco); moderate to severe alcohol use disorder within the past twelve months.

**Table 1:** Demographic, clinical and cannabis use characteristics of study population.

Variable	HIV- (n = 22)	HIV+ (n = 43)	Overall (n = 65)
Sex (% male)	72.2	95.3	87.7
Age (years ± SEM)	47.2 ± 3.5	59.7 ± 1.7	55.5 ± 1.8
Cannabis User (% yes)	45.5	51.2	47.7
Duration of Infection (years ± SEM)		25.9 ± 3.9	
Duration of current antiretroviral regime (months ± SEM)		50.0 ± 8.5	
Global Deficit Score (>0.5 impaired ± SEM)	0.31 ± 0.08	0.46 ± 0.08	0.42 ± 0.06

### 2.2 Neurocognitive assessment

The neurocognitive assessment included a comprehensive battery of neuropsychological tests with appropriate normative data assessing cognitive domains sensitive to HIV and cannabis use. As previously described (65), the Global Deficit Score (GDS) is a composite measure used to assess overall neurocognitive functioning by summarizing performance across multiple cognitive tests. Individual test scores are first converted into demographically corrected T-scores, which are then transformed into deficit scores based on the following scale: T-score ≥ 40 = deficit score of 0; T-score 35 – 39 = deficit score of 1; T-score 30 – 34 = deficit score of 2; T-score 25 – 29 = deficit score of 3; T-score 20 – 24 = deficit score of 4; T-score ≤ 19 = deficit score of 5. Once all individual deficit scores are assigned, they are averaged to produce the GDS, with higher scores indicating greater overall cognitive impairment. This method accounts for both the severity and breadth of impairments across different cognitive domains. A cutoff of GDS ≥ 0.5 was used to indicate NCI (66).

### 2.3 Separation and treatment of monocyte-derived macrophages

**Figure 1:**
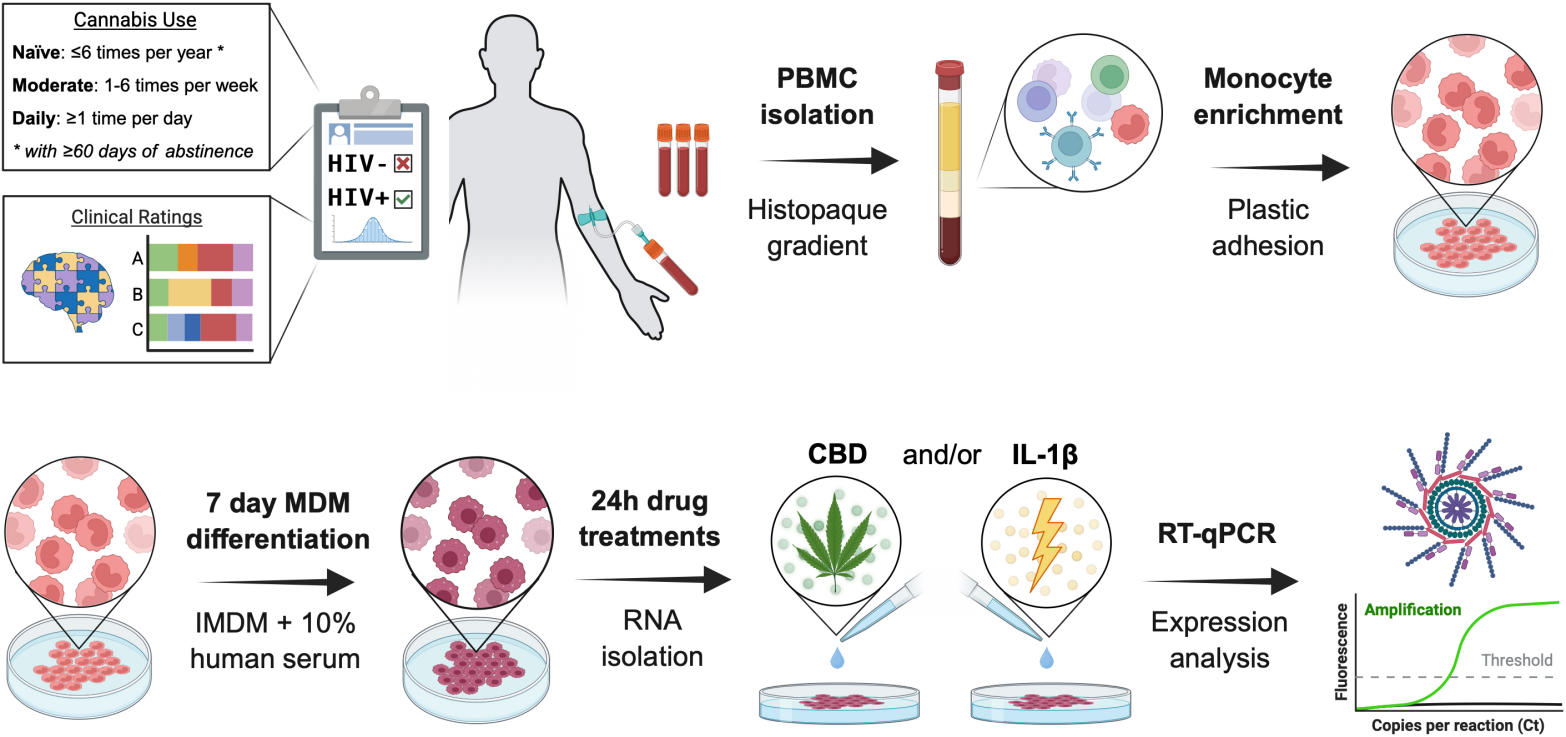
Schematic representation of donor-derived monocyte-derived macrophages’ isolation, culture, and treatment. PBMC, peripheral blood mononuclear cells; IMDM, Iscove’s Modified Dulbecco’s Medium; CBD, cannabidiol; IL-1β, interleukin-1β; Design created with the aid of BioRender.

As illustrated in **Figure 1**, peripheral blood mononuclear cell (PBMC) isolation was performed on donor blood using HISTOPAQUE-1077 (Sigma Life Sciences; #10771) and density gradient centrifugation at 400g for 30 minutes. The PBMC layer was collected, diluted 1:1 with 1X PBS, and centrifuged at 250g for 10 minutes. Cells were washed, centrifuged, and resuspended three times in 1X PBS before resuspension in Iscove’s Modified Dulbecco’s Medium (IMDM; Gibco; #12440053) supplemented with 10% human serum (Millipore Sigma; #H5667) and 1% penicillin/streptomycin (Gibco; #15140122). Automated cell counting was performed on a Countess™ 3 FL (ThermoFisher Scientific; #AMQAF2000) using 0.4% trypan blue solution (Amresco; #K940100ML). Cells were plated in 24-well plates (Corning; #3524) at 400, 000 cells/well for RNA testing or 96-well plates (Thermo Scientific; #164588) at 100, 000 cells/well for immunocytochemistry analyses. Cells were maintained in a humidified incubator at 5% CO2 and 37 °C. Monocytes were isolated via plastic adhesion and non-adherent cells were removed with media exchanges every 2–3 days. After seven days, matured monocyte-derived macrophages were pre-treated for one hour with cannabidiol (Cerilliant Supelco; #C-045; 30 μM) or delta-9-tetrahydrocannabinol (Cerilliant; #T-005; 10 μM) before incubating with IL-1β (Invivogen; #6409-44-01; 20 ng/mL) for 6 hours prior to RNA isolation or 24 hours prior to fixation and immunostaining. Selected concentrations and treatment durations were based on prior studies demonstrating reproducible immunomodulatory effects in primary macrophages while minimizing cytotoxicity (67, 68). All conditions were treated in biological triplicate.

### 2.4 Real-time quantitative polymerase chain reaction

Total RNA was isolated from MDMs with the Qiagen RNeasy Plus Mini Kit (Qiagen; #74136) following kit-provided instructions. RNA was quantified with a Nanodrop 1000 spectrophotometer and reverse transcribed to cDNA using the High Capacity cDNA (Applied Biosystems; #4368814) kit per manufacturer instructions. To quantify the expression of mRNA targets, Taqman (ThermoFisher) probes for *NLRP3* (4331182), *IL1β* (4331182), *IL18* (4331182), and *ACTB* (Applied Biosystems; #4310881E) were incubated with the cDNA. Multiplex relative quantification assays were performed on a QuantStudio 3 Real-Time PCR machine (ThermoFisher). Fold changes were calculated against controls using the comparative Ct method, as previously described (69), and were analyzed in technical duplicates.

### 2.5 Detection and measurement of interleukin-18

Secreted IL-18 protein levels were quantified in cell culture supernatants using a commercially available Human IL-18 ELISA kit (RayBiotech; Norcross, GA; #ELH-*IL18*), according to the manufacturer’s instructions. Samples and standards were processed in duplicate, and absorbance was measured at 450 nm using a Synergy HTX plate reader (BioTek Instruments Inc.; Winooski, VT).

### 2.6 Statistical analysis

Data are presented as mean ± SEM with statistical analyses that include one-way and two-way ANOVA with Cohen’s d and effect size analysis when appropriate. Statistical significance was determined at p < 0.05 for all data, with individual p-values reported when near the significance threshold. All sample sizes and data normalizations are listed in the figure or the figure legends. Data was analyzed on GraphPad Prism 10.3 software (San Diego, CA, USA).

## 3 Results

### 3.1 HIV infection is associated with increased *NLRP3* gene expression

**Figure 2:**
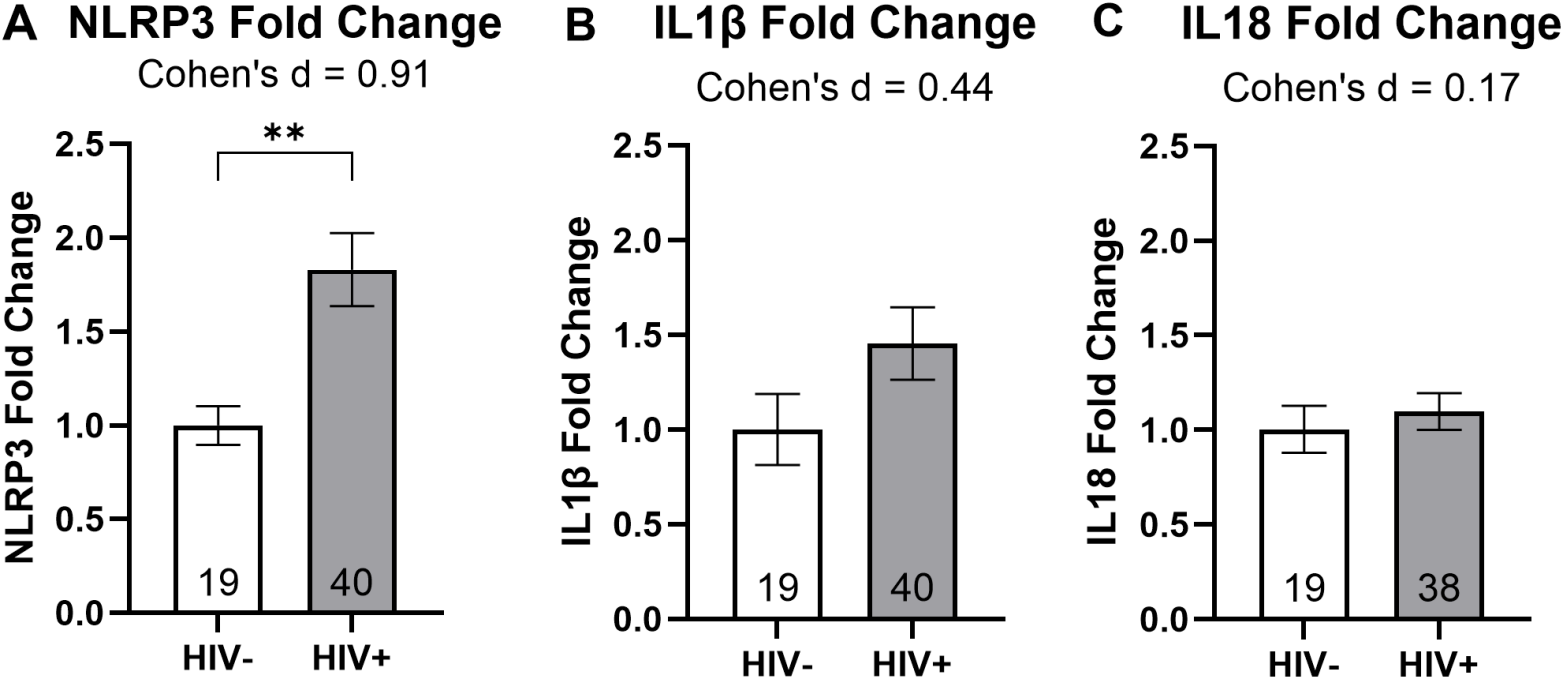
HIV infection is associated with increased *NLRP3* mRNA expression. Relative mRNA expression for **(A)***NLRP3*, **(B)***IL1β*, and **(C)***IL18* grouped by HIV status. Data were analyzed using unpaired t-tests and Cohen’s d for effect sizes; normalized to HIV- MDMs; group size indicated within bars; **p < 0.01.

To examine how *NLRP3*, *IL1β*, and *IL18* mRNA expression was influenced by the presence of the HIV prior to treatment, we divided our RT-qPCR results by HIV status. An unpaired t-test revealed a statistically significant 83% increase in *NLRP3* fold change among PWH (P = 0.006; **Figure 2A**). Further analysis using Cohen’s d indicated a large effect size for the *NLRP3* target gene (**Figure 2A**). Although *IL1β* and *IL18* fold changes appeared elevated in PWH compared to PWoH, the unpaired t-test did not indicate statistical significance for these genes. Cohen’s d analysis showed a medium effect size for *IL1β* and a small effect size for *IL18* when comparing PWH and PWoH (**Figure 2B, C**). These results suggest an association between HIV disease and increased *NLRP3* mRNA expression in MDMs.

### 3.2 Cannabis use is associated with differential regulation of *NLRP3* and downstream *IL1β* and *IL18* gene expression

**Figure 3:**
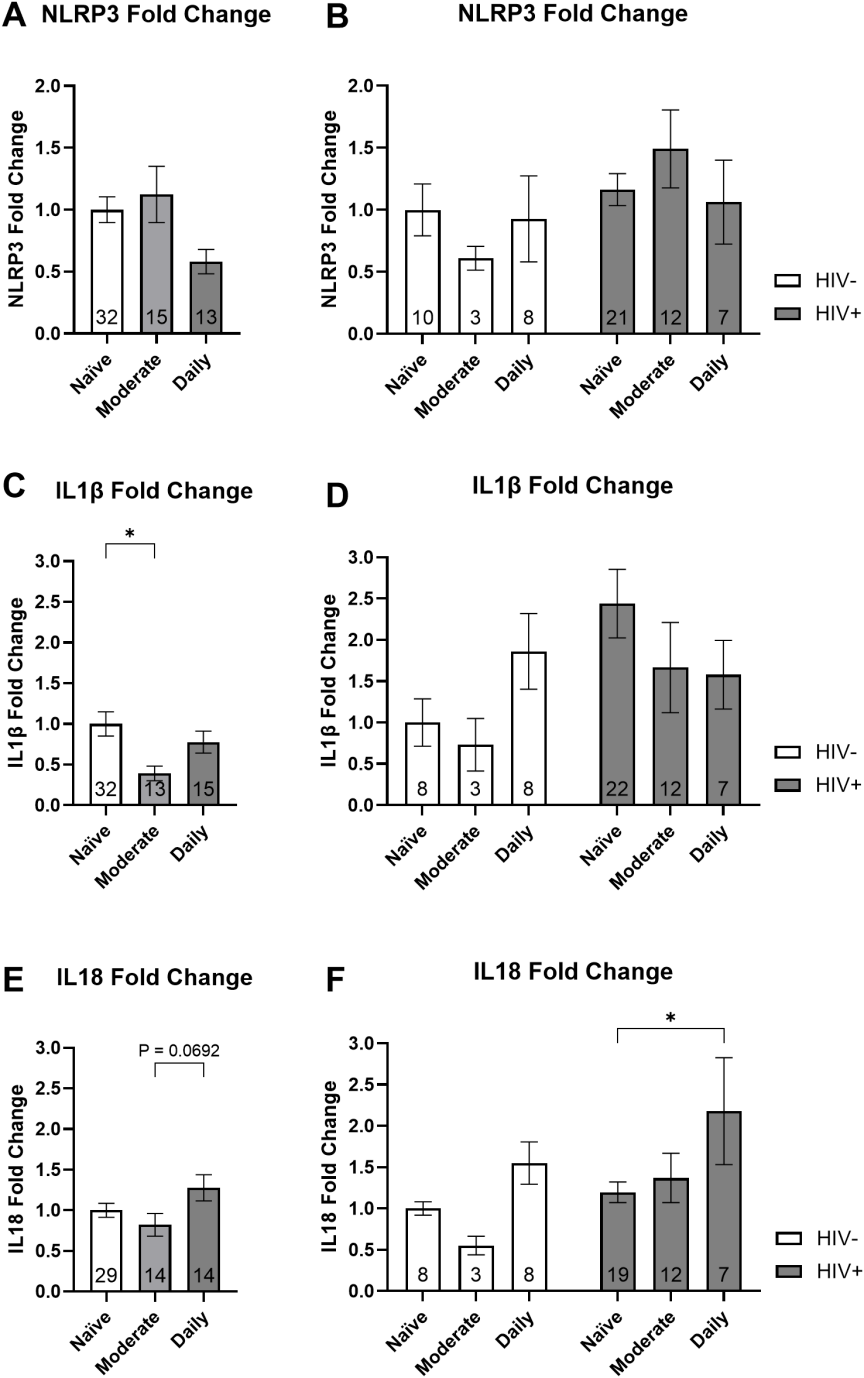
Cannabis use is associated with differential expression of inflammasome-related mRNA: *NLRP3*, *IL1β*, and *IL18*. Relative gene expression stratified by cannabis use for *NLRP3* with the **(A)** donors combined and **(B)** grouped by HIV status, *IL1β* with the **(C)** donors combined and **(D)** grouped by HIV status, and *IL18* with the **(E)** donors combined and **(F)** grouped by HIV status. Data represented as mean ± SEM and analyzed using **(A, C, E)** one-way ANOVA and **(B, D, F)** two-way ANOVA with Holm-Sidak’s multiple comparisons tests; normalized to HIV- naïve MDMs; group size indicated within bars; *p < 0.05, **p < 0.01.

Donor MDMs were then categorized according to their cannabis use patterns to examine associations with *NLRP3* mRNA and downstream *IL1β* and *IL18* mRNA. These comparisons were made in PWH and PWoH combined as well as with the groups separated. We did not observe a significant association between cannabis use groups and *NLRP3* fold change (**Figure 3A**). When stratified by HIV status, there were no significant associations between cannabis use groups and *NLRP3* fold change (**Figure 3B**). Our analysis showed a 61% decrease in *IL1β* mRNA expression from naïve to moderate cannabis users (P = 0.025; **Figure 3C**). When stratified by HIV status, there were no significant associations between cannabis use groups and *IL1β* fold change (**Figure 3D**). Upon analyzing *IL18* mRNA expression in PWH and PWoH we observed a 64% increase in expression with daily cannabis users compared to moderate users, which approached statistical significance (P = 0.06; **Figure 3E**). We observed an 82% increase in *IL18* expression in daily cannabis users relative to naïve users among PWH (P = 0.05; **Figure 3F**). These results suggest that cannabis use is associated with differential regulation of *IL1β* and *IL18* mRNA expression.

### 3.3 The CBD + IL-1β treatment group is associated with decreased *NLRP3* gene expression and increased *IL1β* and *IL18* gene expression

**Figure 4:**
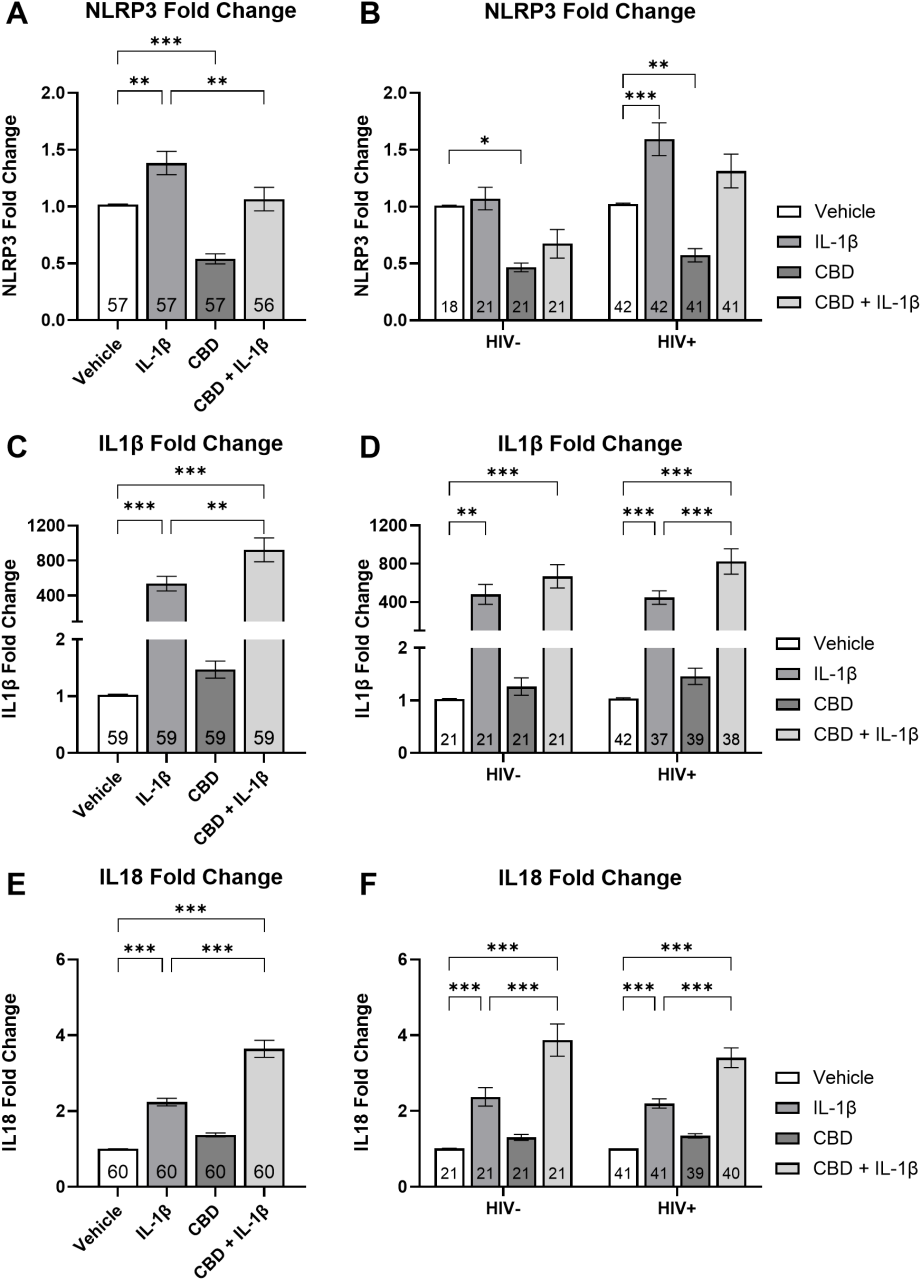
CBD + IL-1β treatment is associated with decreased *NLRP3* mRNA expression and increased *IL1β* and *IL18* mRNA expression in MDMs. Relative *NLRP3* expression stratified by treatment (IL-1β, CBD, and CBD + IL-1β) with donors **(A)** combined and **(B)** grouped by HIV- and HIV +. Relative *IL1β* expression stratified by treatment with donors **(C)** combined and **(D)** grouped by HIV- and HIV +. Relative *IL18* expression stratified by treatment with donors **(E)** combined and **(F)** grouped by HIV- and HIV +. Data represented as mean ± SEM and analyzed using **(A, C, E)** one-way ANOVA and **(B, D, F)** two-way ANOVA with Holm-Sidak’s multiple comparisons tests; normalized to untreated MDMs; Group size indicated within bars; *p < 0.05, **p < 0.01, ***p < 0.001.

MDMs were treated with IL-1β, CBD, and a combination of CBD + IL-1β (C+I), revealing significant differences in *NLRP3* mRNA expression across the treatment groups. MDMs treated with IL-1β showed a 35% increase in *NLRP3* mRNA expression compared to the vehicle group (P = 0.002; **Figure 4A**). In contrast, CBD-treated MDMs exhibited a 47% decrease relative to the vehicle group (P < 0.001; **Figure 4A**). Importantly, the C+I treatment led to a significant 22% reduction compared to IL-1β alone (P = 0.007; **Figure 4A**). When results were stratified by HIV status, statistical significance varied. In HIV-negative MDMs, CBD treatment alone resulted in a 54% decrease from vehicle levels, though no other treatment showed statistically relevant differences (P = 0.03; **Figure 4B**). In HIV-positive MDMs, IL-1β treatment resulted in a 58% increase in expression, while CBD treatment alone produced a significant 44% decrease compared to the vehicle (P < 0.001; P = 0.005; **Figure 4B**). These findings suggest an association between CBD addition and decreased *NLRP3* mRNA expression.

We then examined *IL1β* mRNA expression across treatment groups. Before stratifying by HIV status, the IL-1β-treated group showed a 537-fold increase in *IL1β* expression compared to the vehicle group (P < 0.001; **Figure 4C**). The C+I group exhibited a 924-fold increase over vehicle and a significant 72% increase compared to IL-1β alone (Ps = 0.001; **Figure 4C**). When divided by HIV status, HIV-negative donors showed a 480-fold increase when compared against vehicle (P = 0.01; **Figure 4D**). Furthermore, the *IL1β* expression increased 668-fold in the C+I group compared to the vehicle (P < 0.001; **Figure 4D**). HIV-positive cells treated with IL-1β expressed a 447-fold increase and those treated with C+I expressed an 824-fold increase in *IL1β* mRNA expression relative to vehicle (Ps < 0.001; **Figure 4D**). The C+I treatment further increased expression by 84% over the IL-1β treatment alone (P < 0.001; **Figure 4D**).

Finally, we examined *IL18* mRNA expression. *IL18* mRNA expression in HIV-negative and HIV-positive cells treated with IL-1β showed a 121% increase compared to the vehicle (P = 0.02; **Figure 4E**). Furthermore, those treated with C+I expressed a 260% increase over vehicle (P < 0.001; **Figure 4E**). Cells treated with the C+I combination exhibited an additional significant 61% increase over IL-1β treatment alone (P < 0.001; **Figure 4E**). This pattern persisted when data were stratified by HIV status. In HIV-negative donors, *IL18* expression increased by 134% from vehicle to IL-1β treatment and by 283% from vehicle to C+I (Ps < 0.001; **Figure 4F**). C+I treated HIV-negative MDMs’ *IL18* expression increased 62% from IL-1β (P < 0.001; **Figure 4F**). Among people with HIV, we observed a 112% increase from vehicle to IL-1β treatment and a 237% increase when treated with C+I (Ps < 0.001; **Figure 4F**). C+I treated HIV-positive MDMs’ *IL18* expression increased 55% from IL-1β (P < 0.001; **Figure 4F**). These findings in our *IL1β* and *IL18* cytokine analyses indicate that the combination of CBD and IL-1β is associated with increased inflammatory cytokine mRNA expression in MDMs.

Similar results were observed with THC and THC + IL-1β treatments. In HIV-positive MDMs, THC increased *NLRP3* expression by 42% compared to vehicle, while THC + IL-1β increased expression by 33% compared to vehicle (P < 0.001; P = 0.005; **Supplementary Figure 1A**). However, when comparing the combined treatment to IL-1β alone, no statistically significant differences were observed.

### 3.4 Cannabis use is associated with highest CBD + IL-1β induced changes in *NLRP3*, *IL1β*, and *IL18* gene expression in people with HIV

**Figure 5:**
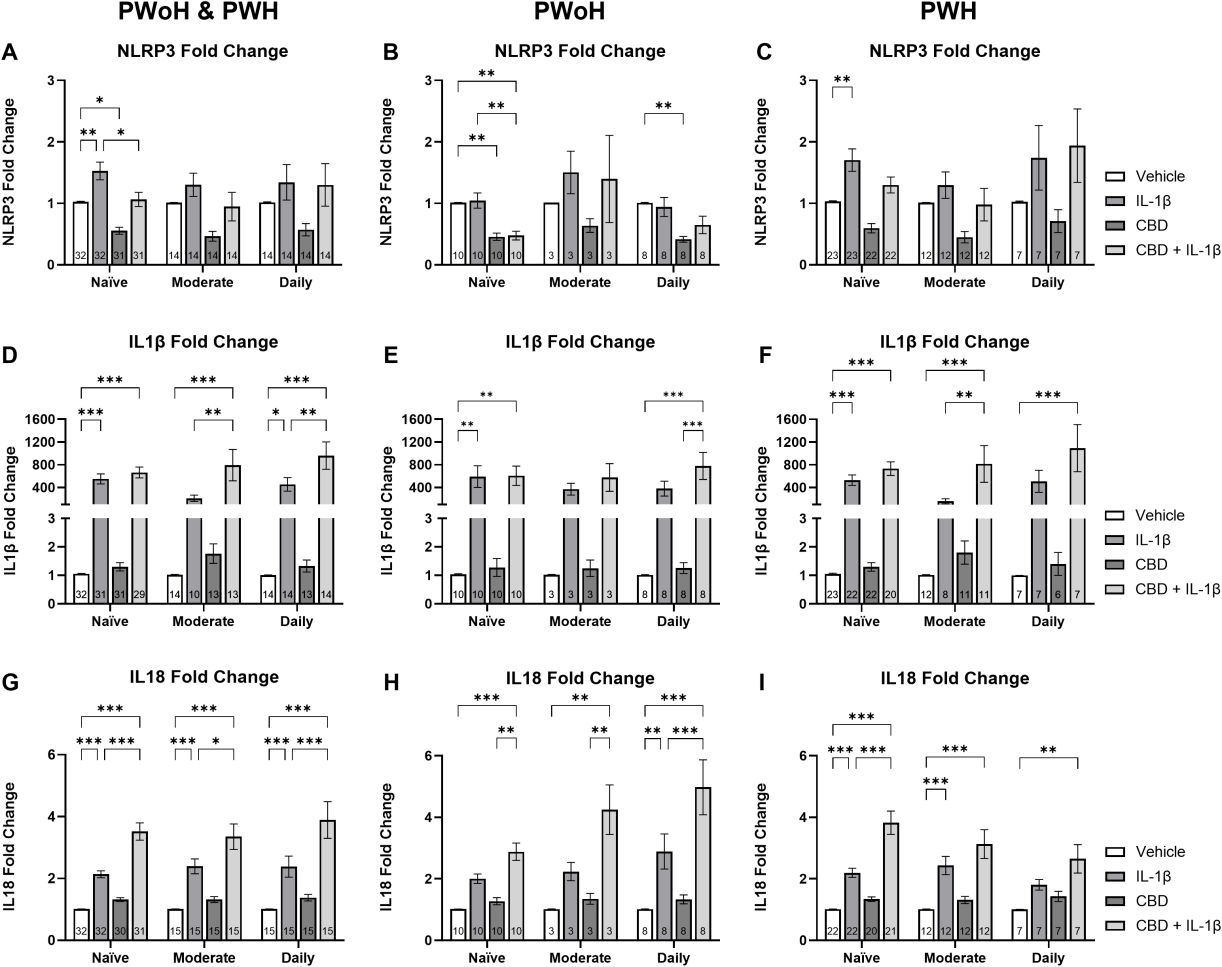
Cannabis use is associated with elevated CBD + IL-1β induced changes in *NLRP3*, *IL1β*, and *IL18* mRNA expression in HIV+ MDMs. Relative *NLRP3* expression stratified by cannabis use patterns (naïve, moderate, and daily) and grouped by **(A)** HIV- and HIV+, **(B)** HIV-, and **(C)** HIV+. Relative *IL1β* expression stratified by cannabis use patterns and grouped by **(D)** HIV- and HIV+, **(E)** HIV-, and **(F)** HIV+. Relative *IL18* expression stratified by cannabis use and grouped by **(G)** HIV- and HIV+, **(H)** HIV-, and **(I)** HIV+. Data represented as mean ± SEM and analyzed using two-way ANOVA with Holm-Sidak’s multiple comparisons tests; normalized to untreated MDMs; Group size indicated within bars; *p < 0.05, **p < 0.01, ***p < 0.001.

To further elucidate the impact of CBD and IL-1β treatment on *NLRP3*-related mRNA expression, we stratified our data by cannabis use patterns and compared across treatment group. Among all naïve cannabis donors, IL-1β-treated cells showed a 50% increase in *NLRP3* mRNA compared to the vehicle while the CBD-treated cells exhibited a significant 47% decrease relative to the vehicle (Ps = 0.01; **Figure 5A**). The combined C+I treatment reduced *NLRP3* mRNA expression by 69% compared to IL-1β alone (P = 0.01; **Figure 5A**). In PWoH naïve cannabis frequency group, CBD treatment decreased *NLRP3* fold change by 43% compared to the vehicle, and the combined treatment further decreased expression by 47% compared to vehicle or (P = 0.002; P = 0.003; **Figure 5B**). Furthermore, the HIV-negative naïve MDMs that received the C+I treatment expressed 55% less *NLRP3* mRNA when compared to the IL-1β treatment (P = 0.002; **Figure 5B**). The HIV-positive naïve MDMs expressed a 67% increase in *NLRP3* expression when treated with IL-1β alone (P = 0.004; **Figure 5C**). Since *NLRP3* mRNA expression was higher in PWH versus PWoH, these results suggest that HIV disease is associated with elevated *NLRP3* mRNA expression.

We next analyzed *IL1β* mRNA expression stratified by cannabis use patterns and compared across treatment group. In the combined group, naïve cannabis users showed a 552-fold increase in *IL1β* mRNA expression following IL-1β treatment (P < 0.001; **Figure 5D**). Similarly, the C+I treatment group exhibited a 664-fold increase over the control (P < 0.001; **Figure 5D**). The combined moderate using group expressed a marked 790-fold increase compared to vehicle and a 277% increase from IL-1β alone (P < 0.001; P = 0.006; **Figure 5D**). Furthermore, the combined daily users treated with IL-1β expressed a 456-fold increase in *IL1β* gene expression (P = 0.02; **Figure 5D**). Similarly, those donors treated with C+I expressed a 960-fold increase over vehicle and a 110% increase over IL-1β alone (P < 0.001; P = 0.008; **Figure 5D**). In HIV-negative donors alone, the naïve group treated with IL-1β demonstrated a 591-fold increase, while the combined C+I group showed a 607-fold increase, both compared to vehicle treatment (Ps = 0.004; **Figure 5E**). HIV-negative daily users exhibited a similar trend with a 780-fold increase from vehicle to C+I treatment reaching statistical significance (P < 0.001; **Figure 5E**). Among PWH alone, naïve users demonstrated a 531-fold increase from vehicle to IL-1β treatment and a 733-fold increase from vehicle to C+I treatment (Ps < 0.001; **Figure 5F**). In PWH moderate cannabis users, the C+I treatment resulted in an 817-fold increase over the control, with an additional 38% increase over IL-1β treatment (P < 0.001; P = 0.009; **Figure 5F**). HIV-positive daily users exhibited a similar trend to the HIV-negative donors with a 1093-fold increase from vehicle to C+I treatment reaching statistical significance (P < 0.001; **Figure 5F**).

When comparing *IL18* mRNA expression by cannabis use patterns across PWH and PWoH, similar trends emerged in all three cannabis use groups. The naïve users *IL18* gene expression increased 112% in the IL-1β treatment and 249% in the C+I treatment compared to vehicle, while the C+I treatment was also 65% higher than IL-1β treatment alone (Ps < 0.001; **Figure 5G**). The naïve users *IL18* gene expression increased 136% in the IL-1β treatment and 232% in the C+I treatment compared to vehicle, while the C+I treatment was also 40% higher than IL-1β treatment alone (Ps < 0.001; P < 0.001; P = 0.02; **Figure 5G**). Daily users *IL18* expression showed the same significant differences with a 136% increase from vehicle to IL-1β, a 285% increase from vehicle to C+I, and notably a 63% increase from IL-1β to C+I (Ps < 0.001; **Figure 5G**). In analyzing PWoH, we found that naïve and moderate cannabis users had 182% and 320% increases from vehicle to C+I, respectively (P < 0.001; P = 0.002; **Figure 5H**). Daily cannabis users’ *IL18* gene expression showed a significant 186% increase from vehicle to IL-1β, a 393% increase from vehicle to C+I, and a 72% increase from IL-1β to C+I (P = 0.002; P < 0.001; P < 0.001; **Figure 5H**). In PWH, naïve cannabis users showed a 117% increase in *IL18* gene expression between vehicle and IL-1β treatments and a larger 278% increase from vehicle to C+I (Ps < 0.001; **Figure 5I**). This group also showed a 74% increase from IL-1β to C+I (P < 0.001; **Figure 5I**). Moderate cannabis users in the HIV-positive group displayed a significant 140% increase between vehicle and IL-1β treatments and a 209% increase from vehicle to C+I treatments (Ps < 0.001; **Figure 5I**). Daily cannabis users showed a 162% increase from vehicle to C+I treatment (P = 0.006; **Figure 5I**). These results, alongside our *IL1β* analysis, suggest that in the context of HIV, increasing cannabis use may be associated with decreasing *IL18* expression and increasing *IL1β* expression.

### 3.5 Quantification of secreted IL-18 from monocyte-derived macrophages stratified by HIV status and cannabis use

**Figure 6:**
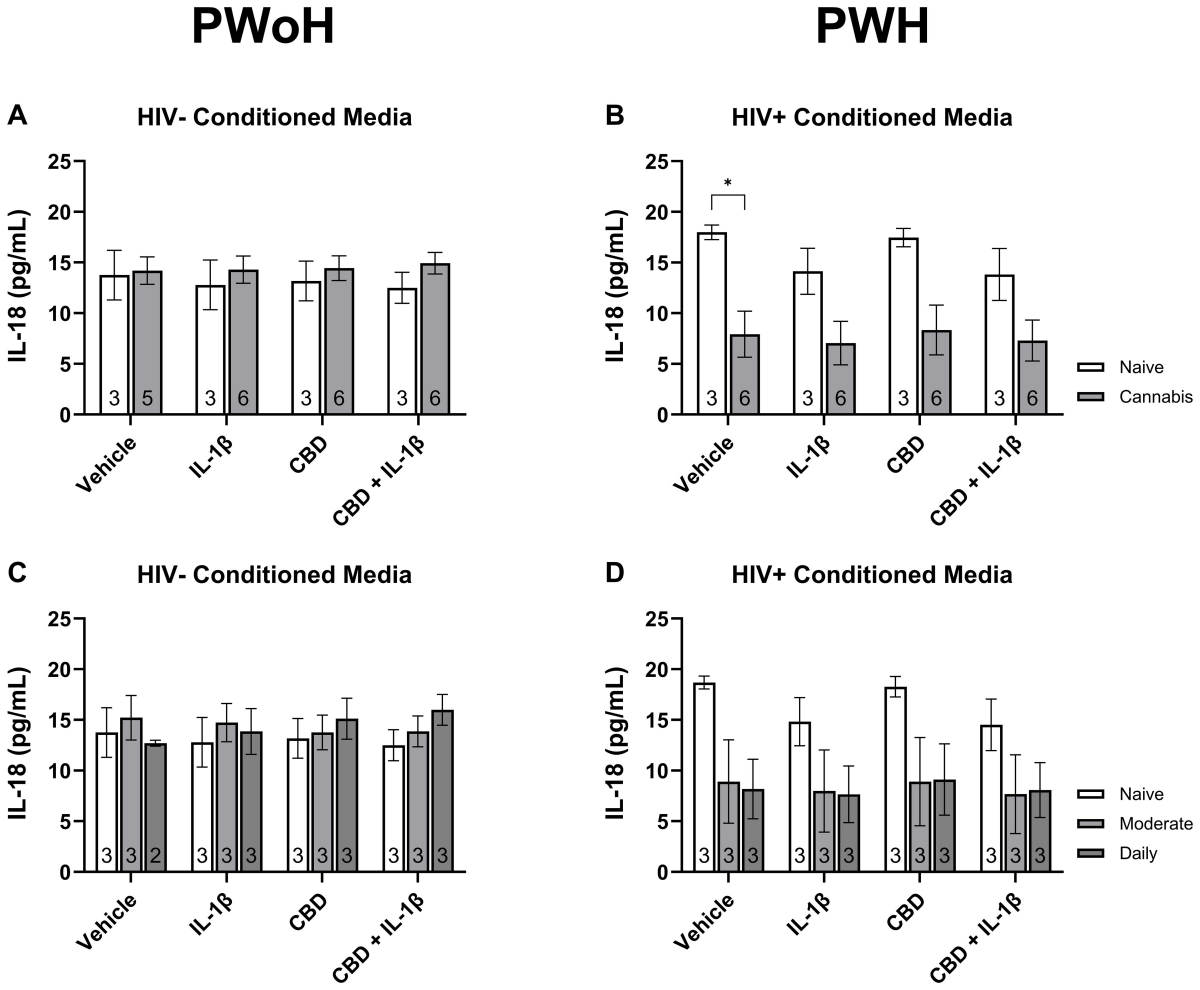
Quantification of secreted IL-18 from monocyte-derived macrophages stratified by HIV status and cannabis use. MDMs from people without HIV [HIV−; **(A, C)**] and people with HIV [HIV+; **(B, D)**] were stimulated with vehicle, IL-1β (10 ng/mL), cannabidiol (CBD; 30 μM), or CBD + IL-1β for 24 hours and IL-18 levels were measured in culture supernatants by immunoassay. **(A, B)** Comparisons between cannabis-naïve and cannabis-using participants. **(C, D)** Comparisons across cannabis use frequency groups (naïve, moderate, daily). Data represented as mean ± SEM and analyzed using two-way ANOVA with Holm-Sidak’s multiple comparisons tests; *p < 0.05.

To complement the transcriptional findings, we quantified IL-18 secretion in MDM supernatants under the same stimulation conditions. As shown in **Figure 6**, IL-18 levels did not significantly differ between cannabis-naïve and cannabis-using donors without HIV (**Figure 6A**) or across cannabis use frequency groups (**Figure 6C**). In contrast, cannabis-using donors with HIV displayed significantly lower basal IL-18 secretion compared with cannabis-naïve donors (P < 0.05; **Figure 6B**), with a similar downward trend across stimulation conditions (**Figure 6D**). These results suggest that cannabis exposure may modulate inflammasome-related cytokine release in PWH, consistent with the transcriptional changes observed.

### 3.6 Correlation of *NLRP3*-related gene expression with immune and neurocognitive parameters

**Figure 7:**
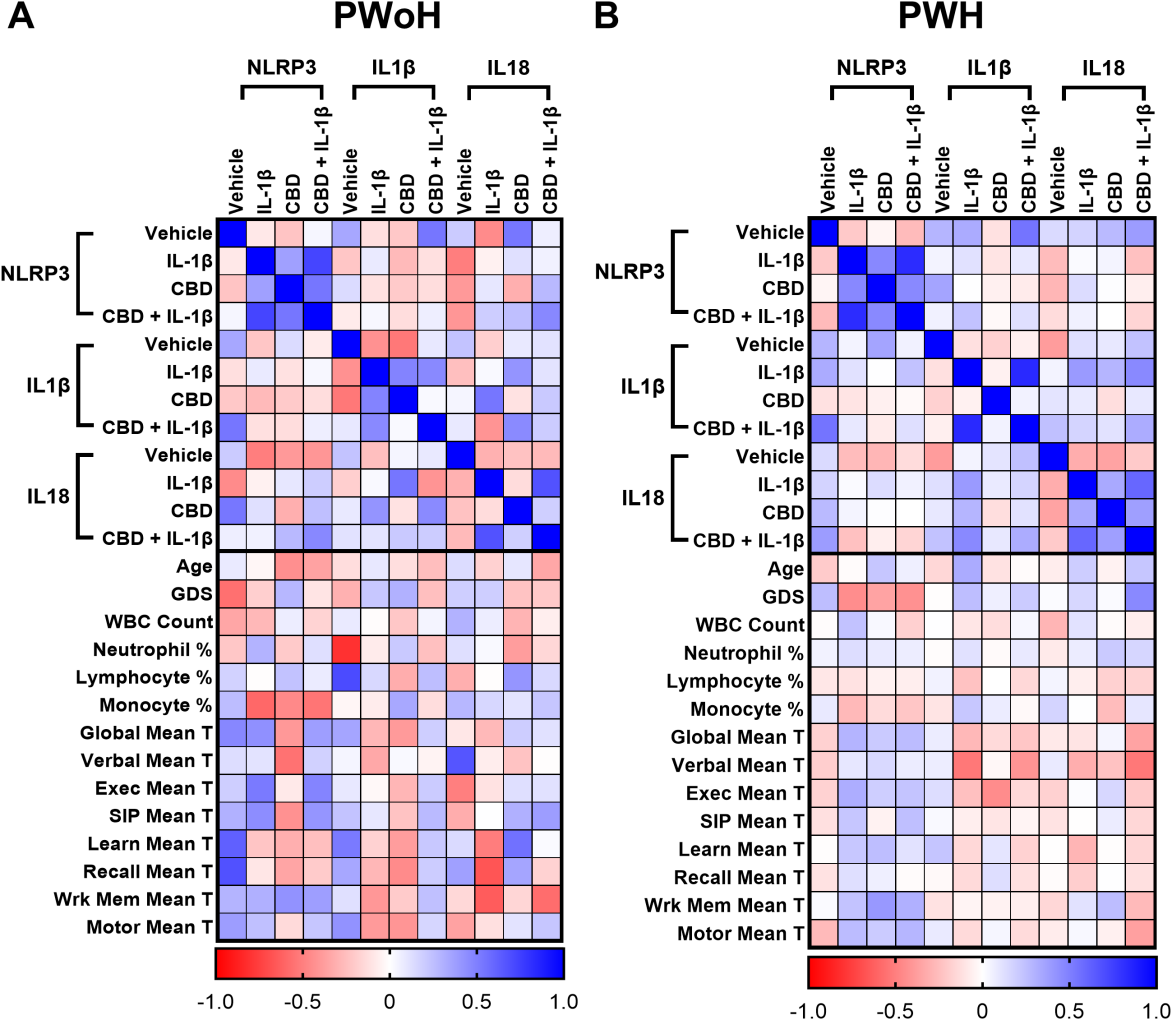
Correlation of *NLRP3*-related gene expression with immune and neurocognitive parameters. Correlation heatmaps comparing the relationship between *NLRP3*, *IL1β*, and *IL18* mRNA expression under different treatment conditions (Vehicle, IL-1β, CBD, CBD + IL-1β) and clinical parameters in **(A)** PWoH and **(B)** PWH. Expression data for each gene-treatment group are correlated with age, global deficit score (GDS), white blood cell (WBC) count, leukocytes percentage (neutrophil, lymphocytes, and monocytes), and domain-specific neurocognitive performance scores (T-scores: global, verbal, executive, speed of information processing (100), learning, recall, working memory, and motor); Pearson correlation coefficients are represented by color intensity; PWoH (n = 19) & PWH (n = 41).

We then generated matrices to assess the impact of HIV status on the correlations between the RT-qPCR results and clinical testing data. These analyses revealed various shifts in correlation, suggesting areas for further investigation. Notably, HIV-negative donors exhibited strong positive and strong negative correlations, many of which were diminished or neutral in HIV-positive donors. Specifically, the global deficit score (GDS) tended to show negative correlations in PWoH, whereas these correlations appeared either positive or neutral in PWH (**Figure 7A, B**).

### 3.7 IL-1β stimulation is associated with reversing the relationship between *NLRP3* gene expression and Global Deficit Score (GDS) in cannabis using PWH

**Figure 8:**
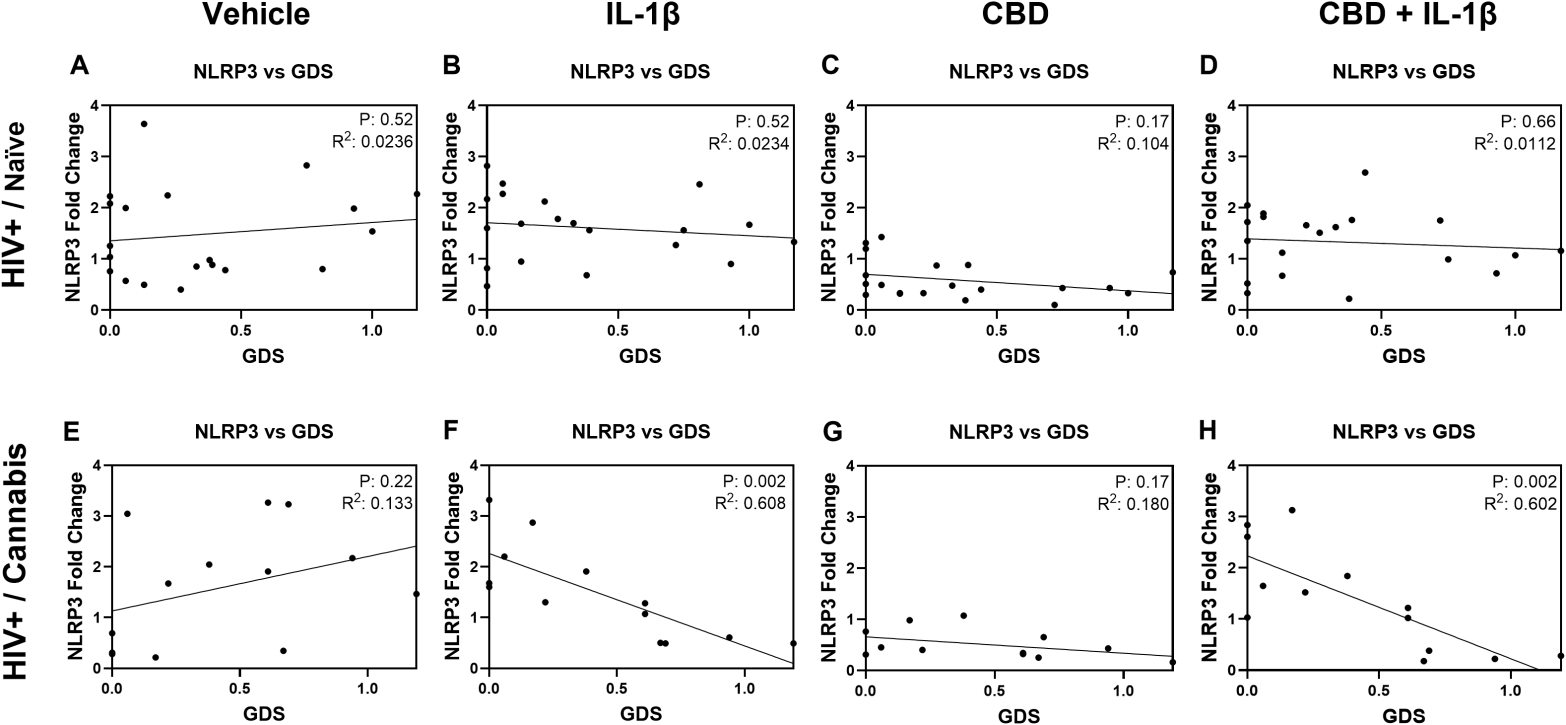
IL-1β stimulation is associated with reversing the relationship between *NLRP3* gene expression and Global Deficit Score (GDS) in cannabis using PWH. Correlation graphs comparing *NLRP3* gene expression and GDS in PWH naïve donors **(A)** before treatment and after **(B)** IL-1β, **(C)** CBD, or **(D)** CBD + IL-1β treatment. Correlation graphs comparing GDS and *NLRP3* gene expression in PWH using cannabis **(E)** before treatment and after **(F)** IL-1β, **(G)** CBD, or **(H)** CBD + IL-1β treatment. Individual significance (p-value) and goodness of fit coefficient (r^2^) determined with Pearson’s correlation coefficient and listed within plots; statistical significance was determined at p < 0.05.

To explore the influence of cannabis use on the relationship between *NLRP3* gene expression and GDS, we generated scatterplots and calculated linear regressions for each treatment group and stratified by cannabis use patterns (naïve and moderate/daily) and HIV status. Among the eight resulting graphs, two showed statistical significance. HIV-positive donors who reported cannabis use exhibited a strong negative correlation, with increased *NLRP3* mRNA expression associated with decreased GDS in MDMs treated with IL-1β (P = 0.002; **Figure 8F**). Similarly, a strong negative correlation was observed in HIV-positive cannabis users, where increased *NLRP3* mRNA expression correlated with decreased GDS in MDMs treated with CBD + IL-1β (P = 0.002; **Figure 8H**). These findings suggest that cannabis use is associated with altering the relationship between *NLRP3* mRNA expression and GDS in HIV-positive individuals who use cannabis.

## 4 Discussion

This is the first study to demonstrate the relationship between HIV disease, cannabis use, and the mRNA expression of *NLRP3*-related genes in MDMs from PWH and PWoH. This research primarily investigates inflammasome priming in MDMs, specifically evaluating the mRNA expression of *NLRP3*-related genes in response to cytokines. Consistent with previous findings, this study demonstrated a significant increase in *NLRP3* mRNA expression in PWH compared to PWoH, consistent with the role of NLRP3 in chronic neuroinflammation in HIV (36, 70–75). The observed differential regulation of *NLRP3* and downstream inflammasome genes like *IL1β* and *IL18* suggests distinct immune mechanisms, with *NLRP3* being more consistently elevated in PWH, while *IL1β* and *IL18* responses varied, possibly reflecting unique regulatory pathways in chronic inflammation (76–78). Additionally, this work sheds light on the significance of inflammasome priming in CBD-treated MDMs from PWH that use cannabis. Specifically, and as described in greater detail below, the effects of cannabis use on inflammasome-related genes varied by HIV status and cannabis use patterns, underscoring the complex role of cannabinoids in modulating inflammatory pathways in HIV disease. These findings, in conjunction with the observed association between *NLRP3* activation and GDS in PWH, indicate the potential for cannabis-induced immune priming to exacerbate neuroinflammatory processes and worsen cognitive outcomes in PWH. As PWH live longer on ART and face a greater risk of age-related neuroinflammatory conditions, these findings point to the need for novel therapeutic strategies targeting NLRP3 to manage neuropathogenesis associated with cognitive dysfunction.

NLRP3 plays a pivotal role in HIV-related neuroinflammation, contributing to persistent immune activation even in ART-treated individuals, where chronic inflammation often persists despite viral suppression (17, 70, 73, 75). Interestingly, certain ART components, such as abacavir, can further activate NLRP3, complicating treatment strategies (79). Activation of NLRP3 triggers the production of pro-inflammatory cytokines such as IL-1β and IL-18, which contribute to the neurodegenerative processes observed in HIV-associated cognitive dysfunction (80). IL-1β, a central pro-inflammatory cytokine, is closely tied to NLRP3 activation and has been implicated in exacerbating neuroinflammatory responses in the CNS (75, 81). In parallel, IL-18, which shares a common processing pathway with IL-1β, also plays a critical role in neuroinflammation and has been linked to both peripheral and central immune activation in PWH (82, 83). Prior studies have suggested that *NLRP3* expression is more consistently elevated in chronic inflammatory states, while IL-1β and IL-18 responses are more variable depending on the specific cytokine milieu and degree of inflammasome activation (78). The activation of NLRP3 in macrophages contributes to sustained immune-mediated damage, as well as heightened expression of pro-inflammatory cytokines like IL-1β and IL-18. The observed differences in *IL1β* and *IL18* expression align with the findings of prior studies, which suggest that NLRP3 activation often results in variable downstream cytokine expression, depending on the local immune environment and degree of activation (36, 78, 84). The consistent upregulation of *NLRP3* in HIV-positive MDMs underscores its central involvement in neuroinflammatory pathways linked to cognitive dysfunction. This evidence reinforces NLRP3’s potential as a therapeutic target for mitigating neuroinflammation and improving outcomes in HIV-associated cognitive impairment.

Inflammasome priming is a crucial aspect of NLRP3 activation, especially in HIV-related neuroinflammation, where viral components serve as potent priming agents (85–87). This priming effect is compounded by the immune-modulatory effects of cannabis, which can either suppress or enhance inflammasome activation depending on the context (88, 89). In the context of this study, priming may contribute to heightened cytokine responses even in the presence of anti-inflammatory agents like CBD. Indeed, the paradoxical observation that CBD co-treatment with IL-1β reduced *NLRP3* mRNA expression while increasing *IL1β* and *IL18* mRNA expression suggests that CBD may differentially regulate the priming and activation phases of the inflammasome. Prior studies have shown that CBD can inhibit NF-κB–dependent transcription of NLRP3 while simultaneously promoting pro-cytokine gene transcription through selective NF-κB modulation or epigenetic mechanisms such as histone acetylation (90–92). Such dual regulation could uncouple inflammasome priming from downstream cytokine expression, helping to explain the divergent transcriptional patterns observed here. Moreover, the differential expression of *IL1β*, *IL18*, and *NLRP3* across cannabis use patterns provides insights into how cannabis influences immune regulation in PWH. Specifically, the heightened expression of *IL18* alongside *IL1β* in daily cannabis users may reflect the primed state of the immune system, which responds more robustly to stimuli, potentially contributing to sustained neuroinflammatory states that worsen neurocognitive outcomes. The complex interplay between viral priming and cannabinoid modulation of immune responses raises important questions about the potential risks of cannabis use in PWH, particularly concerning its impact on chronic inflammation and cognitive outcomes. Understanding this two-step process—priming followed by activation—is essential when evaluating potential interventions targeting chronic inflammation in PWH. The protein-level findings further support the interpretation that cannabis exposure modulates inflammasome activity in HIV-positive individuals. Specifically, reduced basal IL-18 secretion in cannabis users aligns with downregulation of *NLRP3* expression, suggesting a dampening of inflammasome priming and cytokine release. However, due to sample availability, IL-1β secretion could not be reliably measured across all donor groups, representing a limitation of the current study. Future work should employ multiplexed immunoassays to capture the full spectrum of inflammasome effector cytokines, enabling a more comprehensive functional assessment of inflammasome activation.

Cannabis use produced contrasting effects on inflammasome-related gene expression, with moderate use reducing *IL1β* levels, while daily use increased *IL18* expression, particularly in HIV-positive donors. This dual role of cannabinoids aligns with previous research showing that their effects can vary based on dosage, frequency, and cannabinoid type (60, 61, 63, 93). For example, while CB2 activation has been linked to reduced *NLRP3* expression, chronic or high-dose exposure to cannabinoids has been associated with heightened IL-18 production (61, 94–98). This suggests that cannabinoids can initially exert anti-inflammatory effects but may prime the immune system for pro-inflammatory responses over time, particularly in PWH. Interestingly, reduced *NLRP3* mRNA levels following IL-1β exposure were primarily observed in PWH who used cannabis, potentially indicating that cannabis use could exert anti-inflammatory effects in specific contexts, such as when combined with pro-inflammatory stimuli. This finding underscores the potential for cannabis to modulate immune responses differently depending on prior exposure and baseline inflammation, which may explain the observed variability among cannabis-naïve versus cannabis-exposed donors. The protein-level findings further support the interpretation that cannabis exposure modulates inflammasome activity in HIV-positive individuals. Specifically, reduced basal IL-18 secretion in cannabis users aligns with downregulation of *NLRP3* expression, suggesting a dampening of inflammasome priming and cytokine release. However, due to sample availability, IL-1β secretion could not be reliably measured across all donor groups, representing a limitation of the current study. Future work should employ multiplexed immunoassays to capture the full spectrum of inflammasome effector cytokines, enabling a more comprehensive functional assessment of inflammasome activation. Given the complexity of NLRP3 regulation and its interaction with cannabinoids, future studies should also aim to optimize cannabinoid-based interventions to balance anti-inflammatory benefits with potential risks of immune priming.

We also explored whether treatment-specific changes in MDM gene expression were associated with clinical and biological measures. By correlating *NLRP3*, *IL1β*, and *IL18* expression under different stimulation conditions (vehicle, IL-1β, CBD, and CBD + IL-1β) with demographic, immune, and neurocognitive outcomes, we sought to capture inter-individual variability that may not be apparent when stratifying solely by cannabis use groups. This approach provides a framework for identifying treatment-responsive molecular signatures that could inform personalized medicine strategies, particularly in the context of HIV-associated inflammation and cognitive dysfunction. The association between *NLRP3* expression and cognitive impairment, as measured by GDS, further supports NLRP3’s role in HIV-associated NCI (75). Elevated *NLRP3* expression correlated with higher GDS scores, suggesting that inflammasome activation may contribute to cognitive decline in this population. This finding is consistent with studies demonstrating that chronic neuroinflammation, driven by increased IL-1β and IL-18, contributes to neurodegeneration and cognitive impairment in PWH (75, 83, 99). Notably, significant negative correlations were observed between *NLRP3* expression and GDS in the HIV-positive cannabis group following IL-1β stimulation and CBD + IL-1β stimulation, relationships that were absent in HIV-positive cannabis-naïve individuals. These findings suggest that cannabis use modifies the link between inflammasome regulation and neurocognitive performance, potentially reflecting an altered balance between protective and maladaptive immune responses in PWH who use cannabis. The differences in cannabis effects by usage patterns are further complicated by varying administration routes (e.g., smoking, edibles, vaping), which influence cannabinoid bioavailability and immune responses. Nevertheless, CBD’s impact on *NLRP3* expression demonstrated consistent anti-inflammatory effects across both HIV- and HIV+ MDMs, with a notable decrease in *NLRP3* expression. Additionally, the combination of CBD with IL-1β treatment resulted in reduced *NLRP3* expression compared to IL-1β alone, suggesting CBD’s potential to counteract pro-inflammatory stimuli in a primed immune environment. However, CBD’s effects appeared more pronounced among cannabis-naïve donors, indicating that baseline cannabinoid exposure may alter responsiveness to subsequent treatments. To complement our CBD findings, we also examined the effects of THC on inflammasome-related gene expression. As shown in **Supplementary Figure 1**, THC exposure reduced *NLRP3* expression in HIV+ MDMs and modulated *IL1β* and *IL18* expression in patterns partially overlapping with, but also distinct from, those observed with CBD. These results emphasize that CBD and THC regulate inflammasome activity through different mechanisms. Importantly, these findings highlight that the immunological impact of cannabis use in PWH cannot be attributed to a single cannabinoid, and that future studies should consider the combined effects of CBD and THC, which often co-occur in cannabis products and may produce additive or divergent effects on immune regulation. Additionally, exploring the potential of inhibiting NLRP3 activation to reduce inflammasome-driven neuroinflammation presents a promising avenue for therapeutic development. Careful consideration of cannabinoid composition, dosage, and frequency of use will be crucial to developing safe and effective therapies targeting NLRP3 in PWH. Collectively, these findings emphasize the significance of NLRP3 as both a pathogenic factor and a therapeutic target in managing HIV-related cognitive decline.

While this study enhances our understanding of how cannabis use and CBD influence *NLRP3*-related gene expression in PWH, it is crucial to recognize several limitations that may impact the interpretation and generalizability of our findings. Firstly, our analysis focused exclusively on mRNA expression without measuring corresponding protein levels. This limitation was primarily due to the restricted number of MDMs that could be obtained from each donor, which prevented us from performing assays requiring large cell inputs, such as immunoblotting for cleaved caspase-1. Future work with greater sample availability should employ methods that require fewer cells, such as FAM-FLICA staining or multiplex cytokine secretion assays, to functionally validate inflammasome activation. Secondly, the use of MDMs as a model for inflammation restricts our findings to peripheral cells, which may not fully represent the neuroinflammatory processes occurring within the CNS. To further ensure the validity of our MDM model, we monitored conditioned media for viral reactivation. p24 ELISA detection occurred only in control experiments where MDMs were exposed to exogenous HIV (HIV-1_JRFL_), while no p24 was detected in untreated donor-derived MDMs (data not shown), confirming that viral emergence did not occur under our experimental conditions. Furthermore, not measuring the cannabinoid response in perivascular macrophages or microglia is limiting because MDMs are not resident CNS cells. This limitation inhibits an all-encompassing conclusion of brain macrophage’s role in NLRP3-related inflammation from being formed. Moreover, while we demonstrated associations between cannabis use and changes in *NLRP3*-related gene expression, the lack of additional markers to differentiate immune cell subtypes limits the scope of our interpretations. Additionally, our study relied on a cross-sectional design and did not include longitudinal assessments, which are necessary to establish causal relationships between cannabis use, NLRP3 inflammasome activity, and cognitive outcomes in PWH. While we included PWH with varying patterns of self-reported cannabis use, this may not fully capture the diversity of cannabis use behaviors or other confounding factors, such as ART regimens, that could influence *NLRP3* expression and inflammation. We did not include objective measures (e.g., urine toxicology, cannabinoid blood levels) to confirm recent use; such data enhance the reliability of group classifications. Lastly, this study does not account for the specific compositions of cannabis products used by donors, such as the levels of minor cannabinoids or other cannabis-derived compounds, which may contribute to the observed effects. Future studies should aim to include assessments of protein levels, incorporate longitudinal designs, evaluate neuroimmune interactions with other immune cells in the CNS, and explore the roles of broader cannabis-derived compounds in modulating NLRP3 inflammasome activity.

## 5 Conclusion

In conclusion, this study demonstrates a significant role for the NLRP3 inflammasome in chronic neuroinflammation associated with HIV disease, with findings showing increased *NLRP3* mRNA expression in MDMs from PWH. Additionally, the study reveals that cannabis use influences inflammasome-related gene expression, with differential effects on *IL1β* and *IL18* depending on usage patterns and HIV disease status. While these subgroup findings are intriguing, they should be considered exploratory given the small sample sizes. Nevertheless, these results provide translational evidence for the potential of targeting NLRP3 to manage HIV-associated NCI, especially as PWH live longer on ART. The anti-inflammatory properties of CBD, shown by its ability to reduce *NLRP3* expression, offer promising avenues for therapeutic interventions aimed at mitigating neuroinflammation. Future studies with larger and more diverse cohorts will be crucial to further evaluate the effects of different cannabis compositions, including THC/CBD ratios, and to establish the potential risks and benefits of cannabinoid-based therapies in PWH. Additionally, expanding the analysis to include broader inflammasome pathways and other pro-inflammatory cytokines will provide a more comprehensive understanding of immune responses in HIV. Given the need for effective strategies to address neuroinflammation in PWH, these findings support further exploration of NLRP3 inhibitors, including cannabinoids like CBD, to mitigate chronic inflammation and improve cognitive outcomes. Prospective clinical trials are essential to evaluate the efficacy and safety of these interventions, with a focus on modulating NLRP3 activity without compromising immune function. Careful consideration of cannabinoid type, dose, and usage patterns will be critical to developing therapies that mitigate inflammation without exacerbating downstream inflammatory responses.

## Data Availability

The raw data supporting the conclusions of this article will be made available by the authors, without undue reservation.

## Supplementary material

Supplementary Figure THC exposure is associated with increased *NLRP3* mRNA expression in PWH. *NLRP3* gene expression stratified by treatment (IL-1β, THC, and THC + IL-1β) grouped by (A) HIV- and HIV+ with (B) HIV- and (C) HIV+ grouped by cannabis use. *IL1β* gene expression stratified by treatment and grouped by (D) HIV- and HIV+ with (E) HIV- and (F) HIV+ grouped by cannabis use. *IL18* gene expression stratified by and grouped by (G) HIV- and HIV+ with (H) HIV- and (I) HIV+ grouped by cannabis use. Data represented as mean ± SEM and analyzed using two-way ANOVA with Holm-Sidak’s multiple comparisons tests; normalized to untreated MDMs; Group size indicated within bars; *p < 0.05, **p < 0.01, ***p < 0.001.
