# Supplementary figures and images for: Cannabis use is associated with alterations in NLRP3 inflammasome related gene expression in monocyte-derived macrophages from people living with HIV
Source: Front Immunol. 2025 Nov 7;16:1634203. doi: 10.3389/fimmu.2025.1634203 (PMC12634353; doi:10.3389/fimmu.2025.1634203)

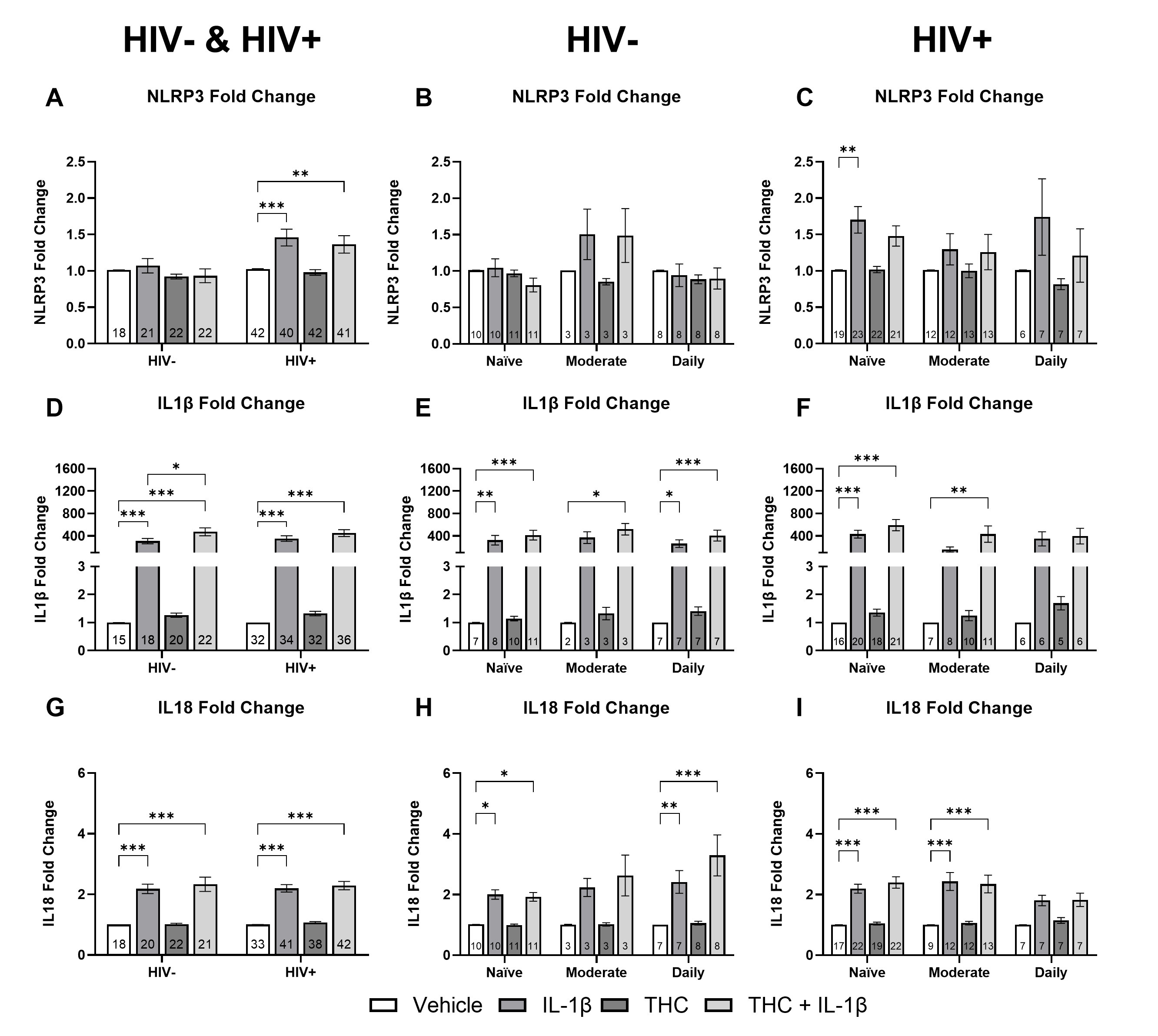

Supplement: Supplementary Figure — THC exposure is associated with increased NLRP3 mRNA expression in PWH. NLRP3 gene expression stratified by treatment (IL-1β, THC, and THC + IL-1β) grouped by (A) HIV- and HIV+ with (B) HIV- and (C) HIV+ grouped by cannabis use. IL1β gene expression stratified by treatment and grouped by (D) HIV- and HIV+ with (E) HIV- and (F) HIV+ grouped by cannabis use. IL18 gene expression stratified by and grouped by (G) HIV- and HIV+ with (H) HIV- and (I) HIV+ grouped by cannabis use. Data represented as mean ± SEM and analyzed using two-way ANOVA with Holm-Sidak’s multiple comparisons tests; normalized to untreated MDMs; Group size indicated within bars; *p < 0.05, **p < 0.01, ***p < 0.001. [file Image1.tif]
